# The Unrecognized Effects of Phosphodiesterase 4 on Epithelial Cells in Pulmonary Inflammation

**DOI:** 10.1371/journal.pone.0121725

**Published:** 2015-04-24

**Authors:** Franziska M. Konrad, Annette Bury, Martin A. Schick, Kristian-Christos Ngamsri, Jörg Reutershan

**Affiliations:** 1 Department of Anesthesiology and Intensive Care Medicine, University Hospital of Tübingen, Tübingen, Germany; 2 Department of Anesthesiology and Intensive Care Medicine, University Hospital of Würzburg, Würzburg, Germany; Chinese Academy of Sciences, CHINA

## Abstract

Acute pulmonary inflammation is characterized by migration of polymorphonuclear neutrophils (PMNs) into the different compartments of the lung, passing an endothelial and epithelial barrier. Recent studies showed evidence that phosphodiesterase (PDE)4-inhibitors stabilized endothelial cells. PDE4B and PDE4D subtypes play a pivotal role in inflammation, whereas blocking PDE4D is suspected to cause gastrointestinal side effects. We thought to investigate the particular role of the PDE4-inhibitors roflumilast and rolipram on lung epithelium. Acute pulmonary inflammation was induced by inhalation of LPS. PDE4-inhibitors were administered i.p. or nebulized after inflammation. The impact of PDE4-inhibitors on PMN migration was evaluated in vivo and in vitro. Microvascular permeability, cytokine levels, and PDE4B and PDE4D expression were analyzed. In vivo, both PDE4-inhibitors decreased transendothelial and transepithelial migration even when administered after inflammation, whereas roflumilast showed a superior effect compared to rolipram on the epithelium. Both inhibitors decreased TNFα, IL6, and CXCL2/3. CXCL1, the strong PMN chemoattractant secreted by the epithelium, was significantly more reduced by roflumilast. In vitro assays with human epithelium also emphasized the pivotal role of roflumilast on the epithelium. Additionally, LPS-induced stress fibers, an essential requirement for a direct migration of PMNs into the alveolar space, were predominantly reduced by roflumilast. Expression of PDE4B and PDE4D were both increased in the lungs by LPS, PDE4-inhibitors decreased mainly PDE4B. The topical administration of PDE4-inhibitors was also effective in curbing down PMN migration, further highlighting the clinical potential of these compounds. In pulmonary epithelial cells, both subtypes were found coexistent around the nucleus and the cytoplasm. In these epithelial cells, LPS increased PDE4B and, to a lesser extend, PDE4D, whereas the effect of the inhibitors was prominent on the PDE4B subtype. In conclusion, we determined the pivotal role of the PDE4-inhibitor roflumilast on lung epithelium and emphasized its main effect on PDE4B in hyperinflammation.

## Introduction

Acute pulmonary inflammation and its more severe form acute respiratory distress syndrome (ARDS) are often seen in critically ill patients leading to hypoxemic respiratory failure with a 40% mortality [1].

In pulmonary inflammation, polymorphonuclear neutrophil granulocytes (PMNs) migrate to the site of inflammation: from the blood to the interstitium of the lung by passing the endothelial barrier—followed by a transepithelial migration from the interstitium into the alveolar space. These two migration steps underlie different regulations [2]. Migration of PMNs is necessary for host defense but excessive PMN migration can result in damaging the epithelial and endothelial barrier and can therefore perpetuate lung injury [3,4].

Out of eleven PDE isoenzymes, PDE4 plays a pivotal role in inflammation [5]. PDE4 degrades exclusively cyclic adenosine monophosphate (cAMP) and 4 subtypes (A-D) are specified; each with a special nonredundant role in the control of cell function [5]. PDE4B and PDE4D dominate in immune cells, especially in PMNs [6]. Studies in knockout mice further revealed different functions of PDE4D and B for inflammation. PDE4B knockout, but not PDE4D, decreased the LPS-stimulated TNFα production in monocytes and peritoneal macrophages [7,8]. On the contrary, Ariga et al. showed evidence that in vitro, PDE4B and PDE4D have complementary effects on PMN migration, one major hallmark of acute pulmonary inflammation [9]. Thereby, PDE4D is mainly responsible for the side effects of the PDE4-blockers and causes emesis and nausea [10]. Because of these, clinical trials with the PDE4-inhibitor rolipram were stopped. PDE4-inhibitors specifically inhibit the enzyme PDE4, including all subtypes [11–13]. The second generation PDE4-inhibitor roflumilast is the first oral obtainable PDE4-inhibitor to treat chronic obstructive pulmonary disease (COPD) associated with a chronic bronchitis and asthma [14,15]. In the first clinical studies, roflumilast was able to improve lung function and to reduce the exacerbation of COPD [16]. The side effects were characterized with a very mild nausea, diarrhea and a light headache [17]. Therefore, we chose roflumilast and the precursor rolipram since roflumilast is already used in humans and increases the clinical impact of our study.

We thought to characterize the effects of the specific PDE4-inhibitors rolipram and roflumilast in terms of PMN migration into the different compartments of the lung, release of chemotactic chemokines, microvascular permeability and thereby focus on the effect of the inhibitors on pulmonal epithelial barrier. Additionally, we characterized the distribution of the PDE4B and PDE4D subtypes on the lung epithelium and their effects on the inflammatory response.

## Methods and Material

### Animals

C57BL/6 male mice were obtained from Charles River Laboratories (Germany) and were 8 to 12 weeks old. All animal protocols were approved by the Animal Care and Use Committee of the University of Tübingen.

### PDE4-inhibitors

After preliminary dose-depending studies, rolipram (1mg/kg) (Sigma-Aldrich; Germany) or roflumilast (500 μg/kg) (LGM Pharma; USA) were applied intraperitoneally [18–20] 1h after LPS inhalation (n = 6).

### Murine model of acute lung injury

As previously described from our lab, 4 to 8 animals inhaled nebulized LPS from salmonella enteritidis (Sigma-Aldrich) (a total of 7 ml, 500μg/ml) in a custom made chamber [21]. LPS inhalation led to an acute pulmonary inflammation with reproducible migration of PMNs into the different compartments of the lung—accumulation intravascular, interstitial, and alveolar migration [22]. Microvascular permeability, chemokine release, and inflammatory changes of receptor expressions are also triggered [23,24]. Control mice were exposed to saline aerosol.

### Immunohistochemistry

Animals were treated with rolipram or roflumilast. Control mice received the solvent. Lungs were prepared as described previously (n = 4) [24].

### In vivo migration assay

24h after LPS inhalation, we determined PMN migration into the different compartments of the lung (intravascular, interstitial, alveolar space) via a flowcytometry-based method as described in detail before [21]. Fluorescent GR-1 (clone RB6-8C5) was injected into the tail vein of mice to mark all intravascular PMNs. The lungs were perfused free of blood to remove nonadherent leukocytes from the pulmonary vasculature. PMNs from the alveolar space were obtained by Bronchial lavage (BAL). Lungs were homogenized and incubated with fluorescent antibodies to CD45 (clone 30-F11) and 7/4 (clone 7/4). Intravascular PMNs were now identified as CD 45+, 7/4+, GR1+, whereas interstitial PMNs were assigned as CD 45+, 7/4+, and GR1- cells. Absolute cell counts were determined in the BAL and lungs (groups without LPS inhalation n = 4; other groups n≥6; different time points of PDE4 inhibitor administration n = 6).

### Nebulization of PDE4-inhibitors

PDE4-inhibitors were dissolved in NaCl and nebulized, so that animals received the same amount of the agents topical (neb) compared to ip (n≥5).

### PDE4B and PDE4D expression and protein level

We determined the expression of PDE4B and PDE4D in lungs of mice by RT-PCR (n≥7). The method was performed as described [23] with the following primers PDE4B (5`-ATC ACC TTG CTG TGG GAT TC -3`and 5`-AAC CAA CCT GGG ATT TTT CC -3`), which detects splice variants of PDE4B a, b, c, d, e, f, g, X1, X2, X3, X5. PDE4D (5`-CAA GTT TCA GGC TGG CTT TC -3`and 5`- GTC CCA TGT GTG ACA AGC AC -3`) amplifies the splice variants 1, 2, 3, 4, 5, 6, 7, 8, 9, X1, X2, X4, X5. Western blots were performed as specified previously (n = 4) [24] and analysed using imageJ, a public program being developed at the National Institutes of Health to officially analyse scientific images.

### Microvascular leakage

To investigate differences between rolipram and roflumilast in terms of their effect on the capillary leakage, Evans blue (20mg/kg; Sigma Aldrich, Steinheim, Germany) was injected into the tail vein 6 h after LPS exposure (n≥6). 30 minutes later, thoracotomy was performed by flushing the lungs with saline into the beating heart, intravascular Evans blue in the lungs was removed, lungs were homogenized, Evans blue was extracted by formamide and the final concentration was determined colorimetrically [21,23].

### Chemokine release

3 hours after LPS inhalation, the release of CXCL 1, CXCL 2/3, tumor necrosis factor-α (TNFα), and interleukin-6 (IL-6) were measured in the BAL of mice (n≥6) [23].

### PDE activity in the lung

To measure the enzyme activity of PDE, lungs of mice treated with rolipram/roflumilast or controls were removed 6.5 h after LPS exposure (control without LPS n = 4; other group n = 6). Lungs were flushed and homogenized with homogenization buffer consisting of 30 mM HEPES and 0,1% Triton X-100 (a total volume of 4 μl per mg lung) [25]. 10 min centrifugation at 13.000 g were followed and 10 μl lung homogenate were mixed with 190 μl PDE-assay buffer consisting of 137 mM NaCl; 2,7 mM KCL; 8,8 mM Na_2_HPO_4_; 1,5 mM KH_2_PO_4_; 1 mM CaCl_2_; and 1mM MgCl_2_. The reaction was started by adding 1 μM cAMP, followed by 10 min at 37°C incubation. 3 min boiling stopped the reaction. After centrifugation at 12.000g for 30 min, the cAMP concentrations—as an indication for PDE activity—in the supernatants were measured using a high-performance liquid chromatography (HPLC). Representative peaks were identified and quantified using a standard curve. PDE activity was calculated reciprocally.

In separate experiments, animals were treated with 3-isobutyl-1-methylxanthine (IBMX) (6.6mg/kg) 1h after LPS ip. Since IBMX is an unspecific PDE-inhibitor, we determined the impact of IBMX and rolipram/roflumilast on the PDE activity to compare the rate of the PDE4 activity with the whole PDE activity of the lung (n = 5).

### In vitro PMN migration

We performed the in vitro transmigration assay of human PMNs through a monolayer of NCI-H441 cells (ATCC, USA) (n≥2) to separate the effects of the PDE4-inhibitors on PMNs and epithelium [23]. Epithelium or PMNs were incubated with rolipram or roflumilast for 60 min at indicated concentrations (n≥4). Until reaching confluence, human epithelial cells were cultivated on inserts of a transwell system (3.0μm pore size, 6.5mm diameter; Costar, Cambridge, MA, USA). Isolated human PMNs (Percoll gradient; GE Healthcare Bio-Sciences AB, Uppsala, Sweden) migrated through the monolayer of epithelial cells along a chemotactic gradient (CXCL2/3; 200ng/ml; Pepro Tech, Hamburg, Germany). By determination of myeloperoxidase were migrated PMNs quantified (absorption length: 405 nm).

### Cytoskeletal remodeling on epithelial cells

We investigated the formation of stress fibers in human epithelial cells to characterize the effect of the PDE-inhibitors on cell remodeling as described [26]. Treatment was blinded for analyses of slides.

### Expression of PDE4B and PDE4D on epithelial cells

H441 cells were incubated with rolipram 100 μM or roflumilast 10 μM for 1 h, and then stimulated with LPS. Rabbit polyclonal anti-PDE4B and goat polyclonal anti-PDE4D were used as primary antibody (Santa Cruz). Images were visualized using a confocal microscope (LSM 510, Meta, Carl Zeiss). Images were analyzed using ZEN 5.0. Treatment was blinded for analyses of slides. Images are representatives of 3 experiments.

To further confirm results from microscopy, we additionally performed western blots from pulmonary epithelial cells and determined the impact of rolipram/roflumilast on PDE4B and PDE4D expression. Western blots were performed as described above.

### Statistical analysis

Data are presented as mean ± SD unless indicated otherwise. Statistical analysis was performed using GraphPad Prism version 5.3 for Windows (GraphPad Software, San Diego, CA, USA). Differences between the groups were evaluated by one-way ANOVA followed by Bonferroni post hoc test. P < 0.05 was considered statistically significant.

## Results

### Time-dependent administration of rolipram and roflumilast

**Fig 1:**
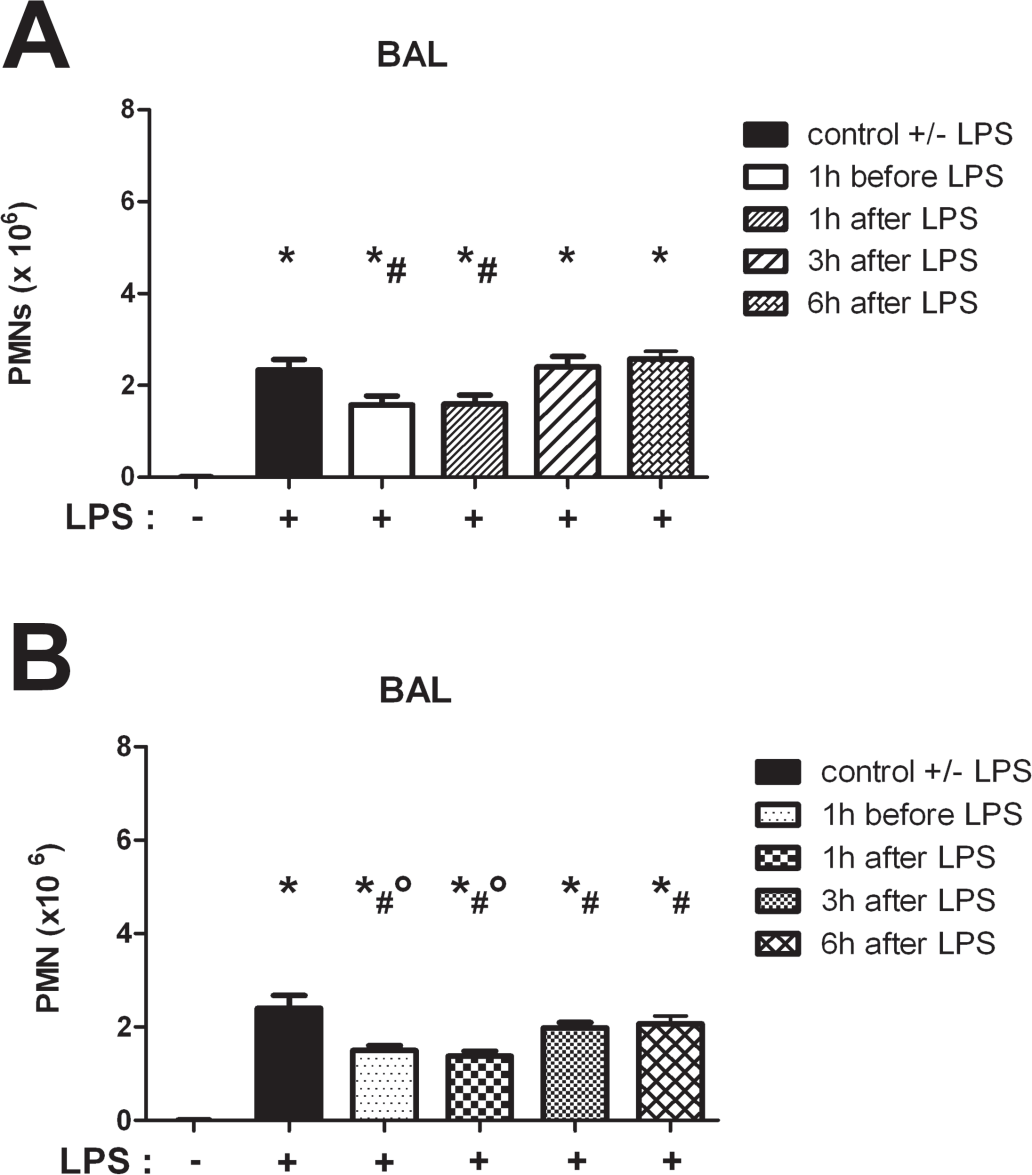
Time optimum for the administration of rolipram (A) and roflumilast (B). The PDE4-inhibitors were given at indicated time points and 24h after LPS inhalation, migration of PMNs into the alveolar space (BAL) was evaluated. Data are presented as mean ± SD; n = 6; *P < 0.05 vs. control without LPS; ^#^P < 0.05 vs. control with LPS; °P < 0.05 vs. 3 and 6h after LPS.

We investigated the time optimum for the administration of rolipram and roflumilast by quantitative determination of PMNs into the alveolar space of mice. Rolipram reduced PMN counts in the alveolar space significantly when administered either 1h before the inflammation or 1h afterwards (1h before LPS: 1.6±0.2x10^6^; 1h after LPS: 1.6±0.2x10^6^ vs. 2.3±0.2x10^6^; P < 0.05), indicating an effect on transepithelial migration (Fig 1A). To increase the clinical impact of our study, we chose 1h after LPS as time point for rolipram administration for all following experiments.

Roflumilast reduced migrated PMNs into the bronchoalveolar lavage (BAL) significantly at any time point, indicating a pivotal role of roflumilast on the epithelium (Fig 1B). PMN counts were even significantly more decreased 1h before and 1h after LPS compared to other time points (1h before LPS: 1.5±0.1x10^6^; 1h after LPS: 1.4±0.1x10^6^; 3h after LPS: 2.0±0.1x10^6^; 6h after LPS: 2.0±0.4x10^6^ vs. 2.4±0.3x10^6^; all P < 0.05). According to the administration of rolipram, we chose to inject roflumilast 1 h after LPS inhalation in all subsequent experiments.

### Immunohistochemistry

**Fig 2:**
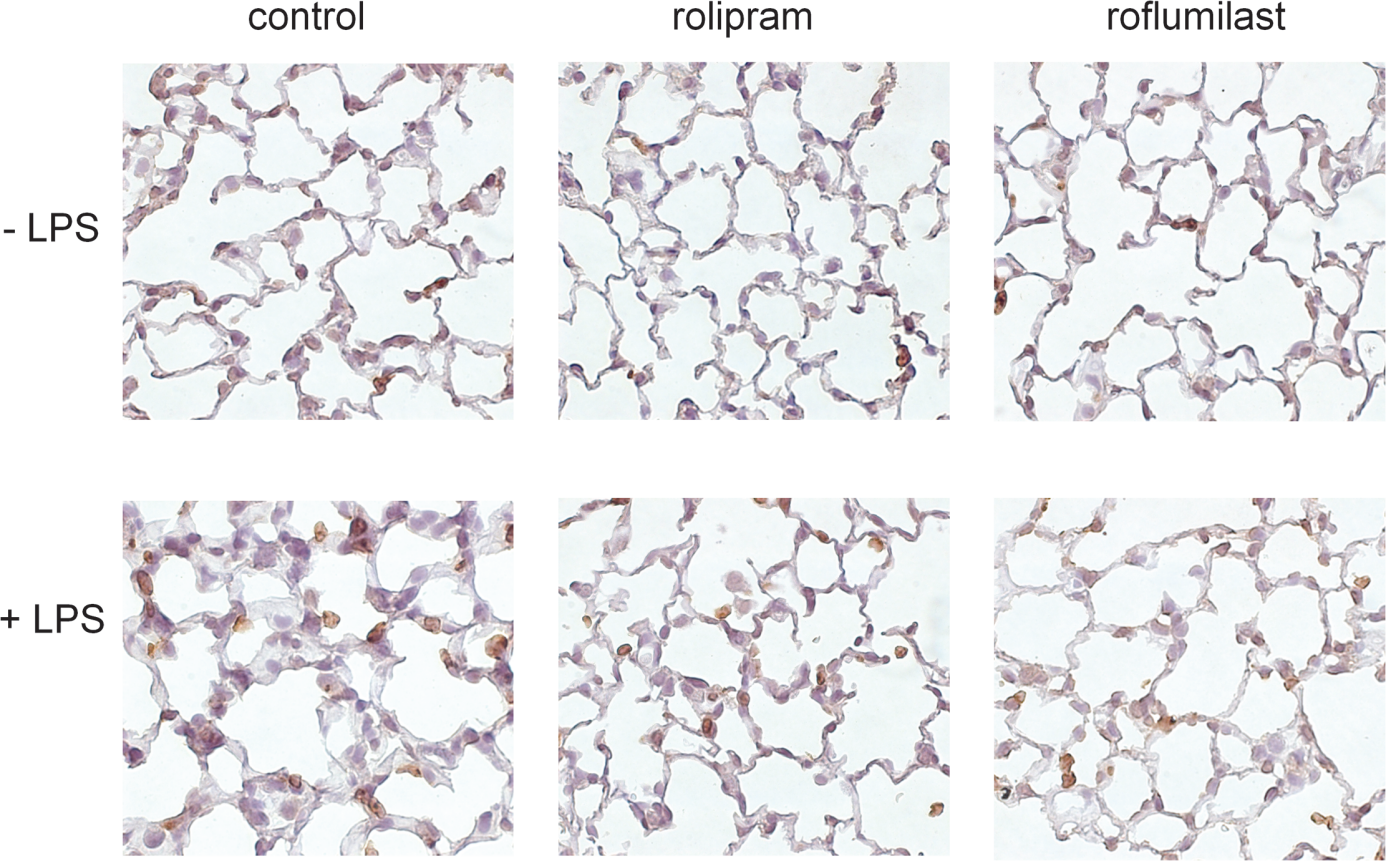
Impact of PDE-inhibitors on PMN infiltration into the lungs identified by immunohistochemistry. Neutrophils were stained with a specific marker and appear brown in histology (rat anti-mouse neutrophil, clone 7/4) (original magnification, x63). Images are representatives of n = 4 experiments. Alveolar septa of the different conditions were measured. Data are presented as mean ± SD; n = 6; *P < 0.05 vs. control without LPS; ^#^P < 0.05 vs. control with LPS; °P < 0.05 vs. rolipram.

PMNs were labeled with a specific antibody so that they appear brown in histology (Fig 2). Without an inflammatory stimulus, the PDE4-inhibitors rolipram and roflumilast did not lead to any changes. LPS inhalation caused a qualitative increase of PMNs into the lung. Alveolar septa were increasingly edematous and thickened (0.4±0.11 vs. 0.14±0.07 mm; P < 0.05) leading to a destroyed lung architecture. Rolipram administration resulted in a reduced migration of PMNs into the lung, alveolar septa were slender (0.2±0.04mm; P < 0.05) and lung architecture restored. Roflumilast exceeded the effects of rolipram with a further reduction of PMN counts, showing an almost healthy lung architecture with slim septa (0.13±0.02mm; P < 0.05). We did not specifically quantify PMNs in histological sections since this procedure is rather inexact and we preferred to perform additional experiments with a flowcytometry-based method in the following.

### PDE4-dependent PMN migration into the different compartments of the lung

**Fig 3:**
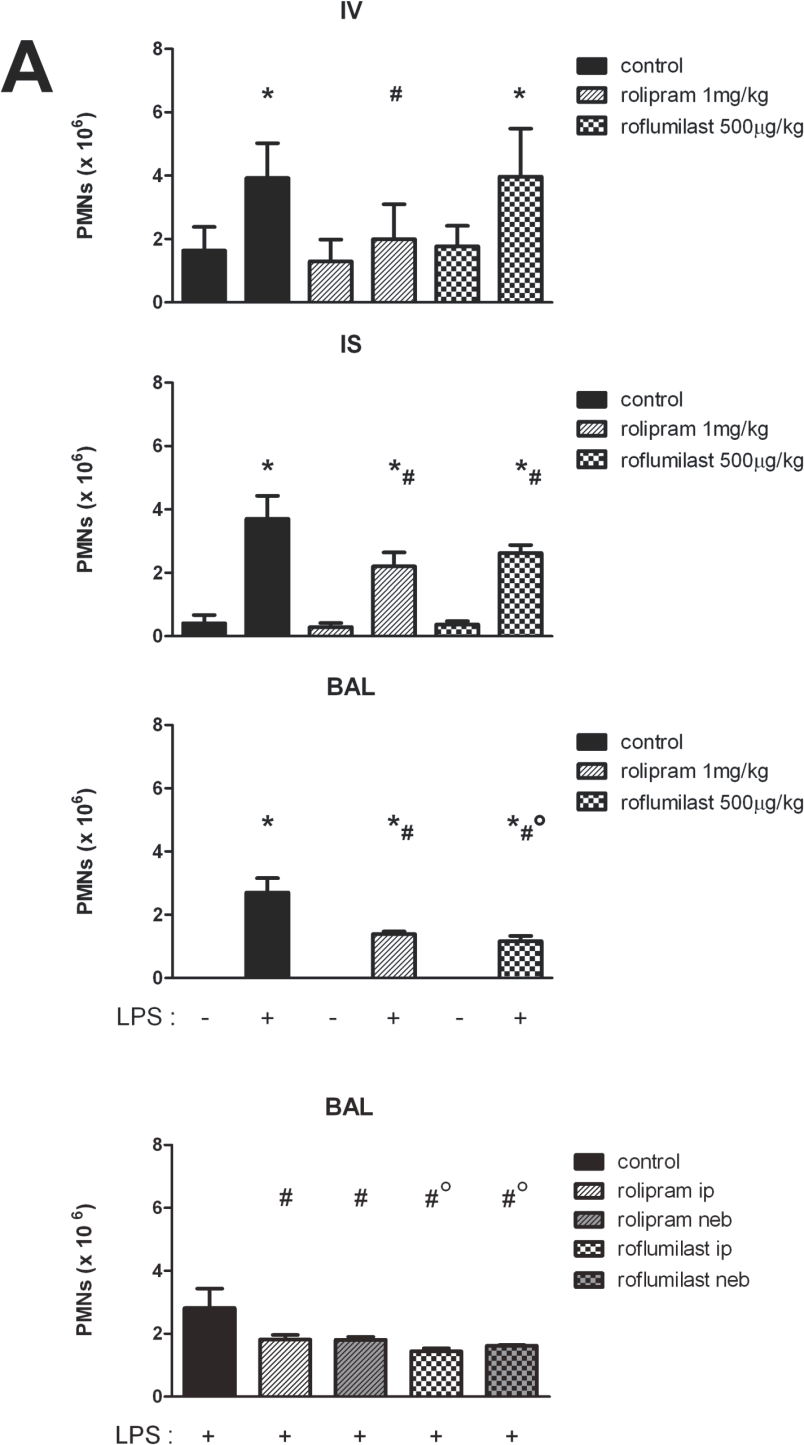
Effect of rolipram and roflumilast on PMN migration into the lung. PDE4-inhibitors were injected and migration of PMNs into the different compartments of the lung without and with LPS (IV = intravascular; IS = intersitital; BAL = alveolar space) quantified (**A**). In additional experiments, the effects of nebulization the PDE4-inhibitors on PMN migration were analyzed (ip = intraperitoneally, neb = nebulized) (**B**). Data are presented as mean ± SD; figure A: n = 4 without LPS; n = 8 with LPS; figure B: n≥4; *P < 0.05 vs. control without LPS; ^#^P < 0.05 vs. control with LPS inhalation; °P < 0.05 vs. rolipram ip.

To further quantify PMN influx into the different compartments of the lung—intravascular, interstitial, and the alveolar space, we performed the in vivo PMN migration assay. Therefore, PMNs were marked with specific antibodies and assigned to the different lung compartments by flow cytometry. Rolipram or roflumilast alone did not alter PMN migration without an inflammatory stimulus in any compartment (Fig 3A). After LPS inhalation, PMNs attached to the endothelium in the intravascular compartment rose significantly (3.9±1.1x10^6^ vs. 1.6±0.7x10^6^; P < 0.05). Rolipram prevented any increase at all (1.8±1.0x10^6^; P < 0.05), whereas roflumilast did not alter intravascular PMN counts (4.0±1.5x10^6^). In the interstitium of the lung, LPS caused an increase of migrated PMNs in all groups (all P < 0.05), but to a significantly lesser extend in the PDE4 inhibited groups (rolipram: 2.3±0.5x10^6^; roflumilast: 2.6±0.3x10^6^ vs. 3.7±0.7x10^6^; all P < 0.05). The inflammation augmented PMN counts in the alveolar space in all groups (P < 0.05). Both PDE4-inhibitors had a strong effect on transepithelial migration, but roflumilast outplayed the effect of rolipram (rolipram: 1.5±0.5x10^6^; roflumilast: 1.2±0.2x10^6^ vs. 2.7±0.5x10^6^; P < 0.05), confirming our findings from immunohistochemistry.

### Nebulization of the PDE4-inhibitors

To further determine the clinical impact of rolipram and roflumilast for the treatment of ARDS, the PDE4-inhibitors were applied topically by nebulization (Fig 3B). Ip and topical administration of roflumilast reduced PMN counts in the BAL significantly more compared to rolipram (roflumilast ip: 1.4±0.1x10^6^; roflumilast neb: 1.6±0.0x10^6^ vs. rolipram ip: 1.8±0.2x10^6^; rolipram neb: 1.8±0.1x10^6^; all P < 0.05), highlighting again the strong effect of roflumilast on the epithelium and emphasizing the opportunity of topical administration with the possibility of decreasing side effects.

### The effect of the PDE4-inhibitors on chemokine release

**Fig 4:**
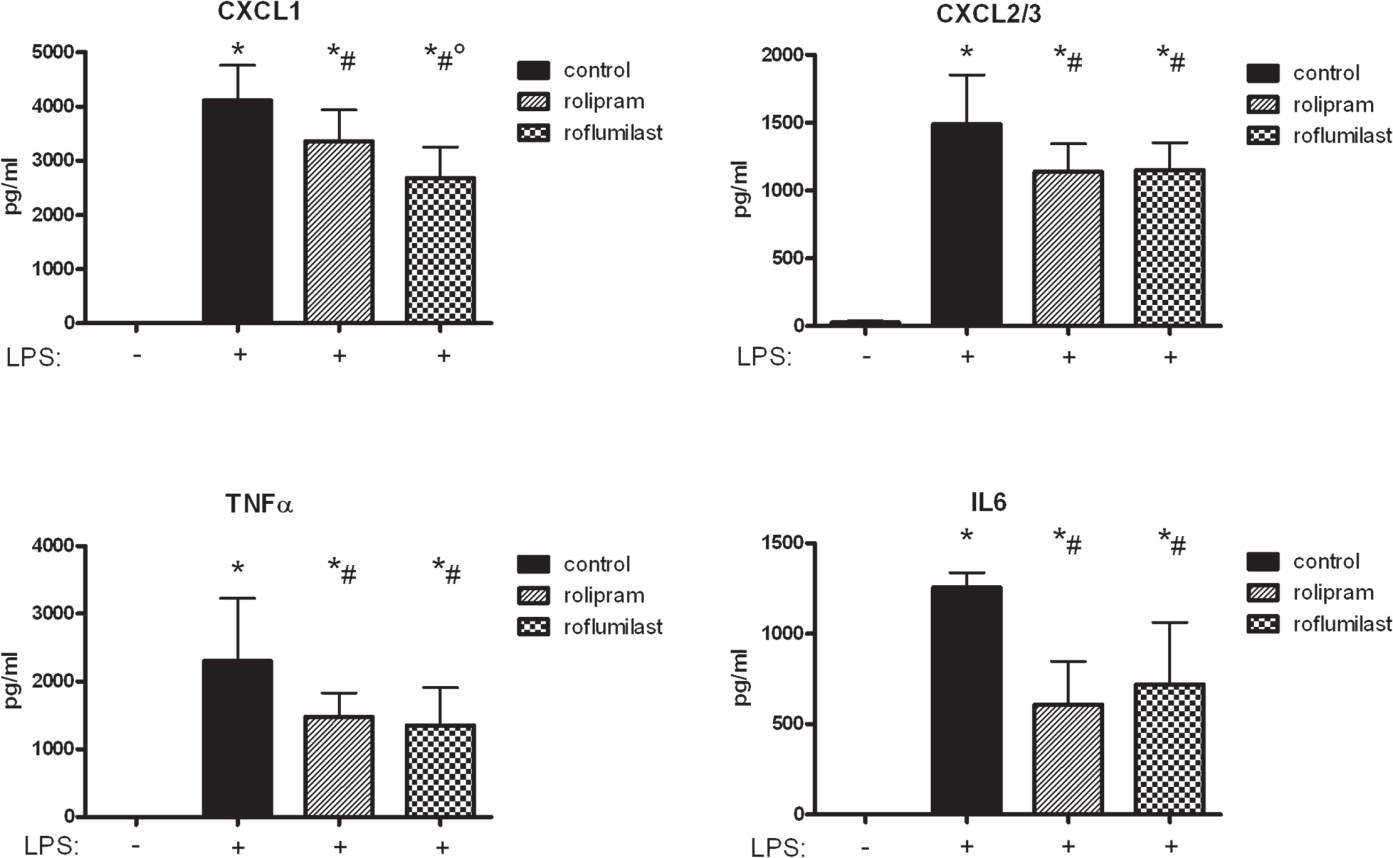
Chemokine levels in the BAL are reduced by rolipram and roflumilast. LPS inhalation increased all chemokine levels and the administration of the PDE4-inhibitors decreased this effect. Data are presented as mean ± SD; n = 4 without LPS; n≥6 with LPS; *P < 0.05 vs. control without LPS; ^#^P < 0.05 vs. control with LPS inhalation; °P < 0.05 vs. rolipram.

We determined chemokine concentrations in the BAL since they are the attractants for PMNs to migrate transepithelial. Without LPS inhalation, neither rolipram nor roflumilast altered chemokine release compared to control and are therefore displayed as one bar in the figure (Fig 4). LPS inhalation caused a significant rise of the chemokines CXCL1, CXCL2/3, TNFα, and IL6. PDE-inhibitors significantly decreased CXCL1, CXCL2/3, TNFα, and IL6 to a comparable amount. Roflumilast diminished CXCL1—the PMN chemoattractant secreted by the epithelium—even further compared to rolipram (2676±571 vs. 3358±576 pg/ml; P < 0.05), confirming our findings from the in vivo migration assay with lowest PMN counts after roflumilast treatment and emphasizing an outstanding role of roflumilast on the epithelium.

### The impact of PDE4-inhibitors on microvascular permeability

**Fig 5:**
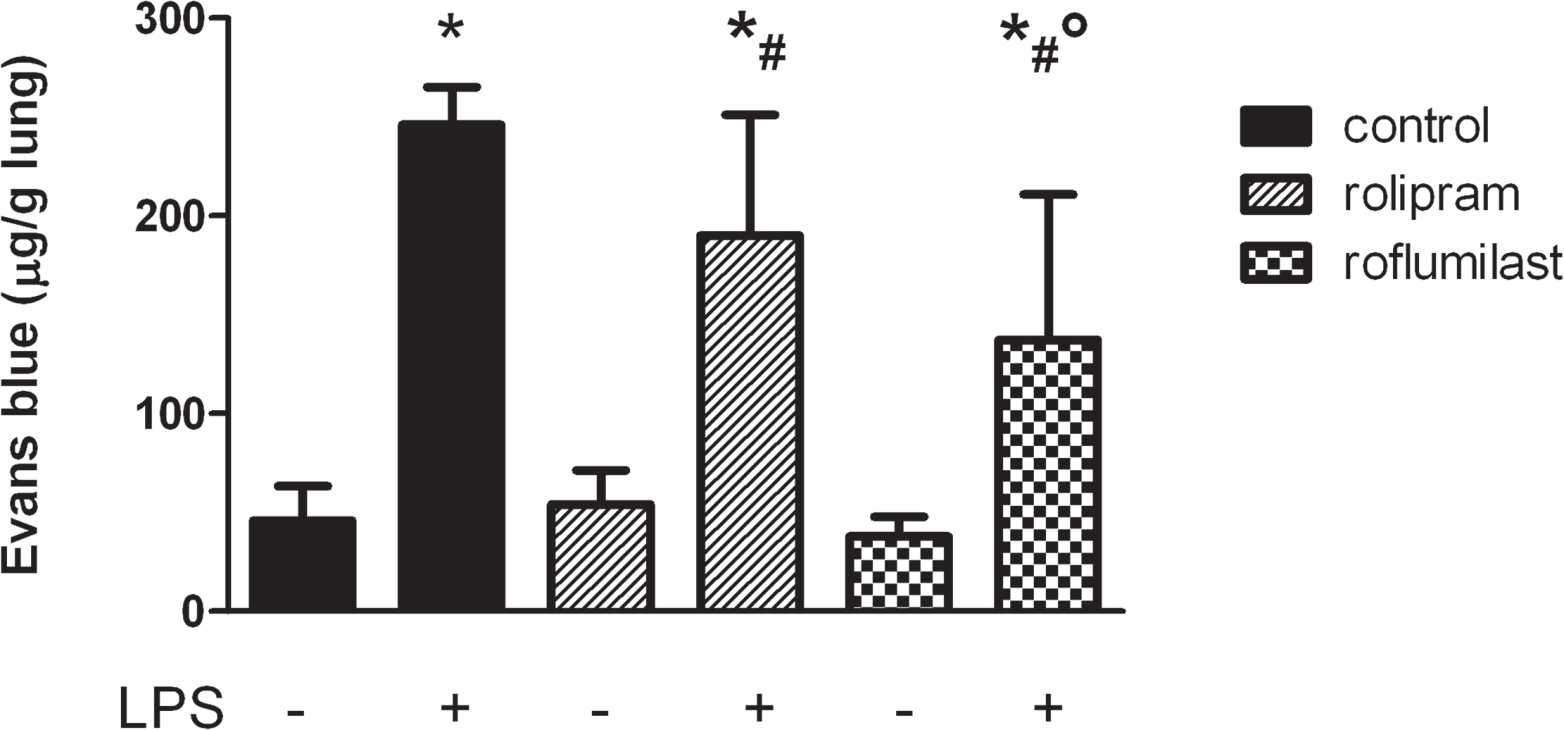
Microvascular permeability was attenuated by PDE4-inhibitors. 6h after LPS inhalation, the capillary leakage was assessed by Evans blue extravasation. Data are presented as mean ± SD; n = 6; *P < 0.05 vs. control without LPS; ^#^P < 0.05 vs. control with LPS inhalation; °P < 0.05 vs. rolipram.

We determined microvascular permeability by means of Evans blue extravasation technique. Without LPS inhalation, the PDE4-inhibitors did not alter Evans blue extravasation (Fig 5). LPS caused a significant increase of the capillary leakage in all groups (LPS: 246±19; rolipram: 190±61; roflumilast: 137±73 vs. 45±18 μg/g lung; all P < 0.05). Both PDE4-inhibitors reduced microvascular permeability significantly, but roflumilast demonstrated a significantly stronger effect on stabilization the capillary barrier.

### Effects of rolipram and roflumilast on the expression of PDE4B and PDE4D in the lung

**Fig 6:**
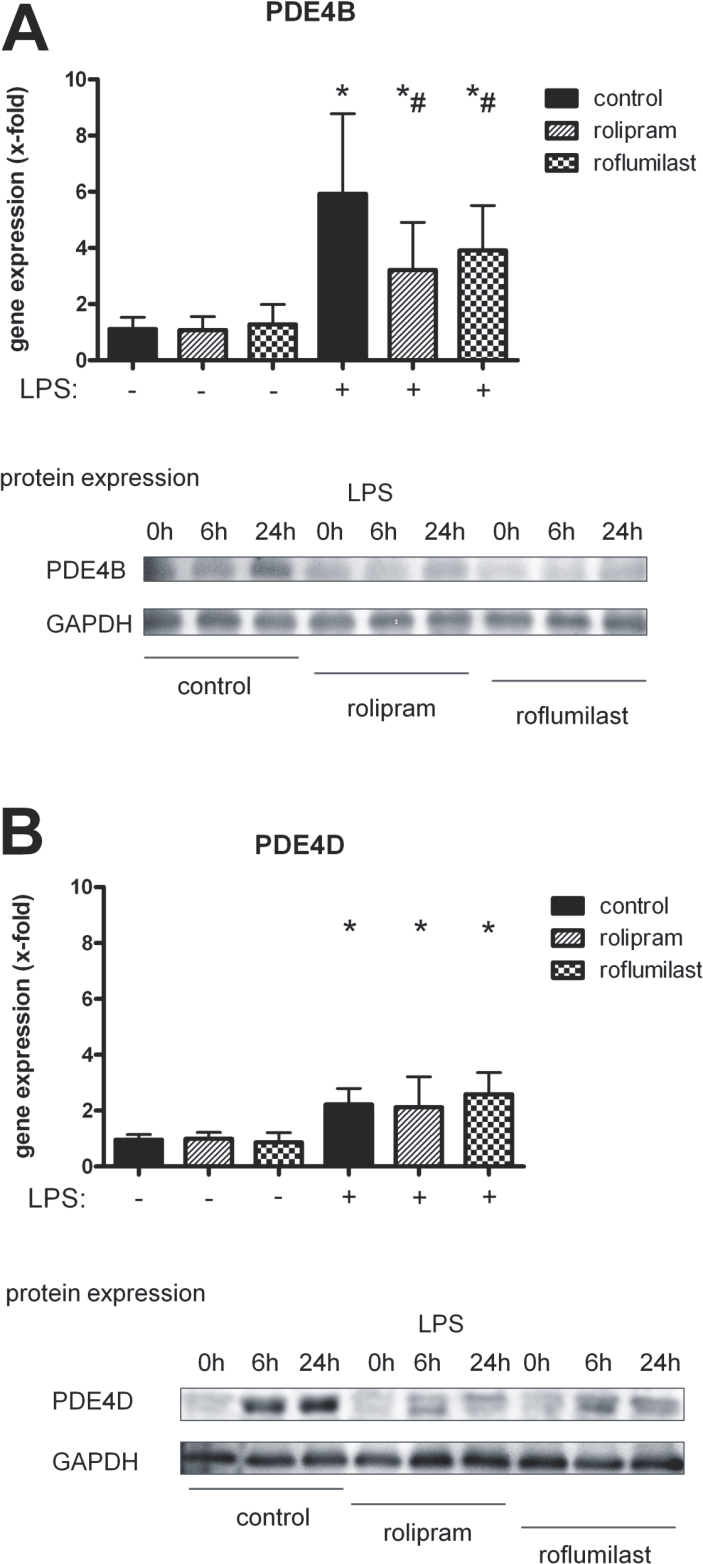
Gene expression and protein levels of PDE4B and PDE4D in lungs of mice. The impact of rolipram and roflumilast on transcription and translation of PDE4B (**A**) and PDE4D (**B**) was determined in the lungs of mice. Data are presented as mean ± SD; n = 6 without LPS; n≥7 with LPS; representative Western blot analyses of four independent experiments (for whole western blots see S1 Fig); *P < 0.05 vs. control without LPS; ^#^P < 0.05 vs. control with LPS inhalation.

LPS inhalation caused a significant increase of the expression of the inflammatory PDE4B (5.9±2.9 vs. 1.1±0.4; P < 0.05) (Fig 6A). Rolipram and roflumilast decreased the expression about a comparable amount (rolipram: 3.2±1.7; roflumilast: 3.9±1.6; all P < 0.05), reflecting their anti-inflammatory potential in terms of inflammation. These findings were confirmed on protein level via western blots (the whole blot is shown in S1 Fig).

LPS also increased PDE4D, but to a lesser extent (2.2±0.6 vs. 1.0±0.2; P < 0.05) (Fig 6B). Neither rolipram nor roflumilast had influence on the expression of the enzyme, which is held responsible for the emetic effects of the inhibitors [10]. On protein levels, the PDE4-inhibitors reduced PDE4D protein but roflumilast less compared to rolipram, showing a presumably temporary effect.

### PDE4 activity

**Fig 7:**
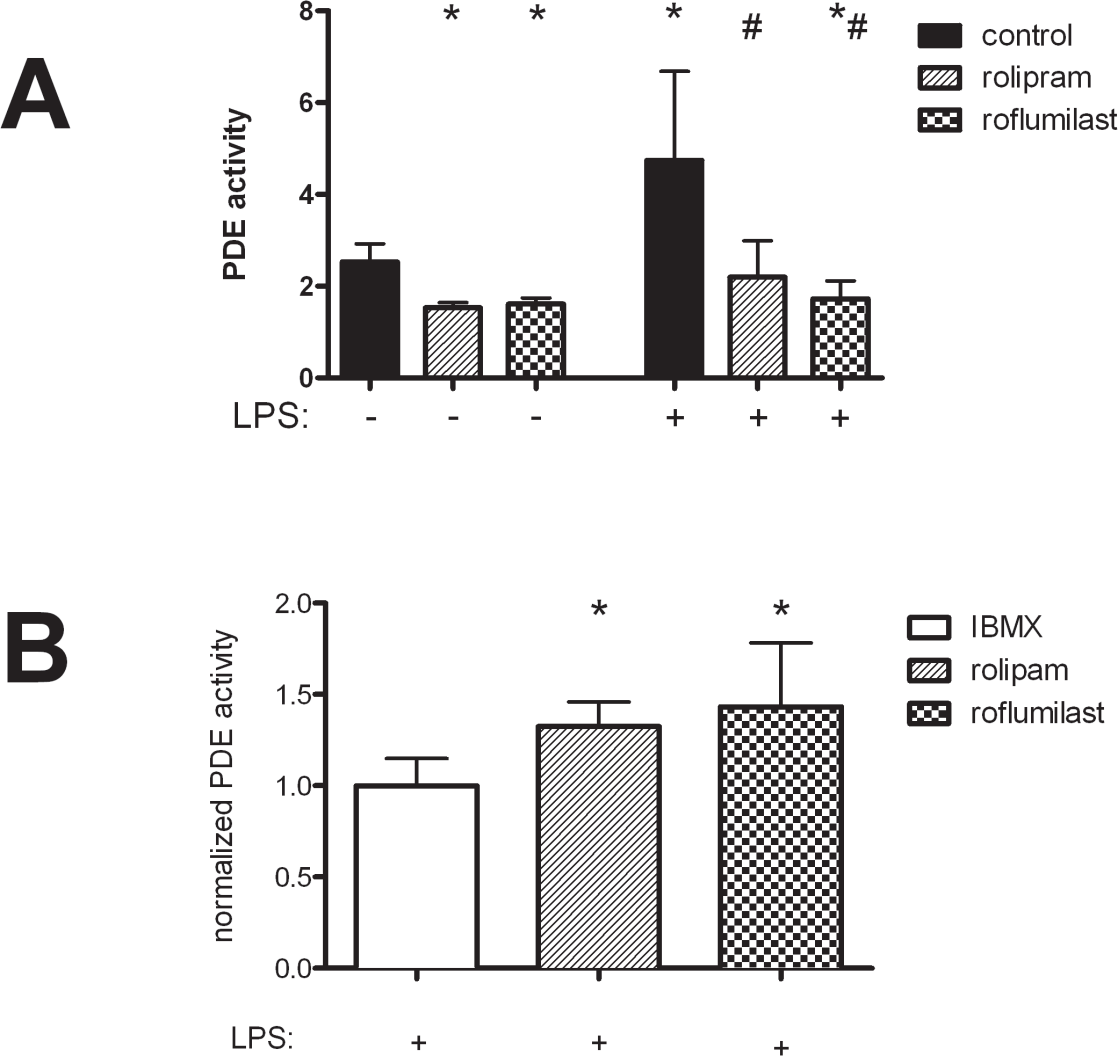
The effect of inflammation and rolipram/roflumilast on PDE activity (A). Mice were treated with roflumilast or rolipram and inflammation induced by LPS inhalation. Lung homogenates were prepared for PDE4 activity. PDE4-inhibitors decreased the activity of the enzyme even without inflammation. Data are presented as mean ± SD; groups without LPS n≥4; groups with LPS n = 6; *P < 0.05 vs. control without LPS; ^#^P < 0.05 vs. control with LPS inhalation. The impact of the unspecific PDE-inhibitor 3-isobutyl-1-methylxanthine (IBMX) on the PDE activity compared to the PDE4-inhibiors (B). Mice inhaled LPS and were treated with PDE4-inhibiors or IBMX. Data are presented as mean ± SD; n = 5; *P < 0.05 vs. IBMX group.

PDE4-inhibitors specifically increase cAMP levels by blocking the subtypes of the enzymes. We determined PDE4 activity by using a PDE4 activity assay, where changes of cAMP levels in the lungs of mice were measured and the activity reciprocally calculated (Fig 7A). PDE-inhibitors significantly reduced the activity even without an inflammatory stimulus (rolipram: 1.53±0.11; roflumilast: 1.61±0.14 vs. 2.53±0.39; all P < 0.05). LPS inhalation significantly increased the activity of the PDE (4.75±1.93; P < 0.05) and rolipram inhibited the enzyme (2.20±0.79; P < 0.05). But roflumilast reduced PDE4 even more effective to values comparable with the roflumilast group without LPS (1.72±0.4; P < 0.05), reflecting the higher anti-inflammatory potential of roflumilast in terms of PMN migration, chemokine levels and microvascular permeability.

PDE activity after rolipram and roflumilast were both significantly higher compared to the global PDE-inhibitor IBMX (rolipram: 1.3±0.1; roflumilast: 1.4±0.4 vs. 1±0.2; all P < 0.05) (Fig 7B). Roflumilast and rolipram inhibit PDE4 activity 28% less compared to IBMX. Thereby, IBMX is additionally an unspecific but effective adenosine receptor antagonist [27], which leads to additional changes in cAMP-levels not only correlated with PDE activity. Nevertheless, PDE4 seems to play a predominant role in the lungs in terms of inflammation.

### In vitro transmigration assay

**Fig 8:**
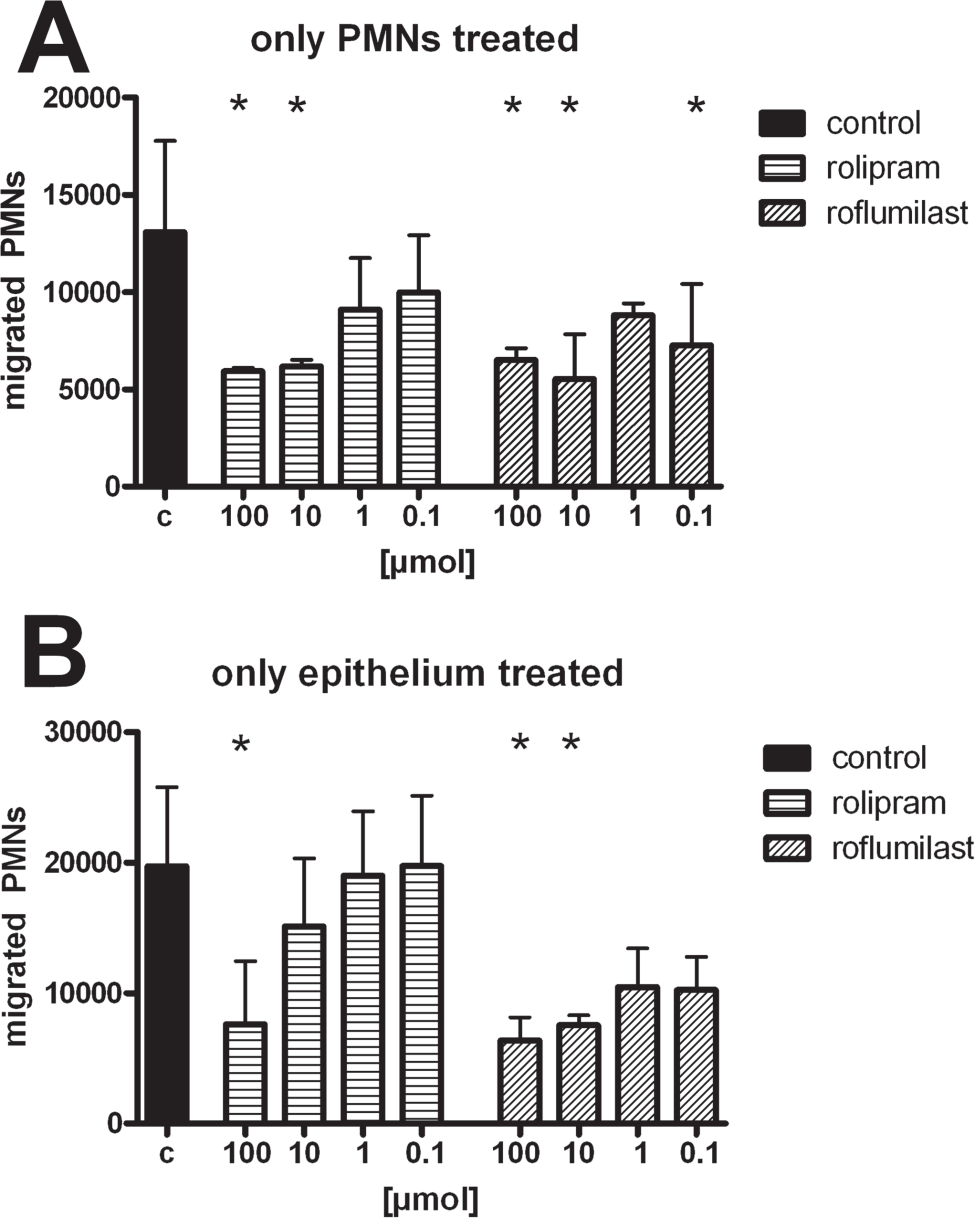
In vitro transmigration assay of human PMNs through a pulmonary epithelial monolayer. PMNs or epithelium were treated with rolipram/roflumilast and migration of PMNs through human epithelium measured. Migration of PMNs was initiated through the chemokine CXCL2/3 in all wells. Data are presented as mean ± SD; n≥4; *P < 0.05 vs. control.

In this assay, isolated human PMNs migrate along a chemotactic gradient through a monolayer of pulmonary epithelial cells. With the in vitro transmigration assay it is possible, to separate the impact of the PDE4-inhibitors on PMNs and on the epithelium. Treated PMNs at certain concentrations migrated significantly less through a monolayer of epithelium (Fig 8). The exclusive treatment of the epithelium was also effective in curbing down PMN migration, whereas roflumilast was still effective at lower concentrations, highlighting a direct pivotal role of the PDE4-inhibitors on stabilization the epithelium (all P < 0.05).

### Cytoskeletal remodeling

**Fig 9:**
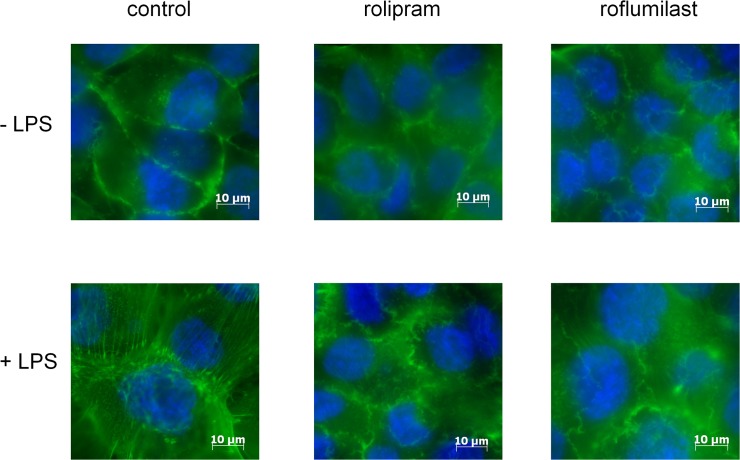
The impact of PDE4-inhibitors on cytoskeletal remodeling. LPS-induced formation of F-actin (green) in human pulmonary epithelial cells. Rolipram and roflumilast reduced cytoskeletal remodeling, whereas the effect was more intense in roflumilast treated cells. Images are representatives of three experiments with similar results (original magnification, x63).

To evaluate the effect of the PDE4-inhibitors on cytoskeletal remodeling as one critical parameter of pulmonary barrier function, we stained F-actin in pulmonary epithelial cells (Fig 9). The PDE-inhibitors did not alter cytoskeletal remodeling without an inflammatory stimulus. LPS-induced stress fibers, which are an essential requirement for a direct migration of PMNs into the alveolar space. Roflumilast and also, but less prominent, rolipram reduced these stress fibers, indicating their pivotal role on epithelial barrier function and confirming our in vitro transmigration assay.

### PDE4B and PDE4D distribution on epithelial cells

**Fig 10:**
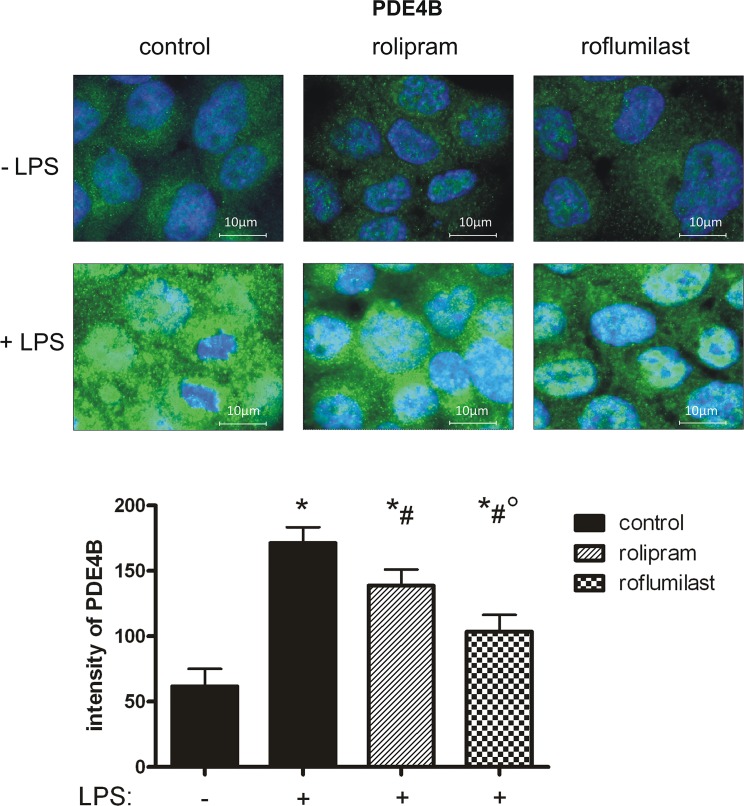
The effect of LPS and rolipram/roflumilast on PDE4B protein expression in epithelial cells. Human pulmonary epithelial cells were pretreated with rolipram/roflumilast and the influence of LPS on the expression of PDE4B (green) (**A**) investigated. Images are representatives of four experiments with similar results (original magnification, x63). Data are presented as mean ± SD; n = 3; *P < 0.05 vs. control group without LPS inhalation, ^#^P < 0.05 vs. control with LPS; °P < 0.05 vs. the rolipram group.

**Fig 11:**
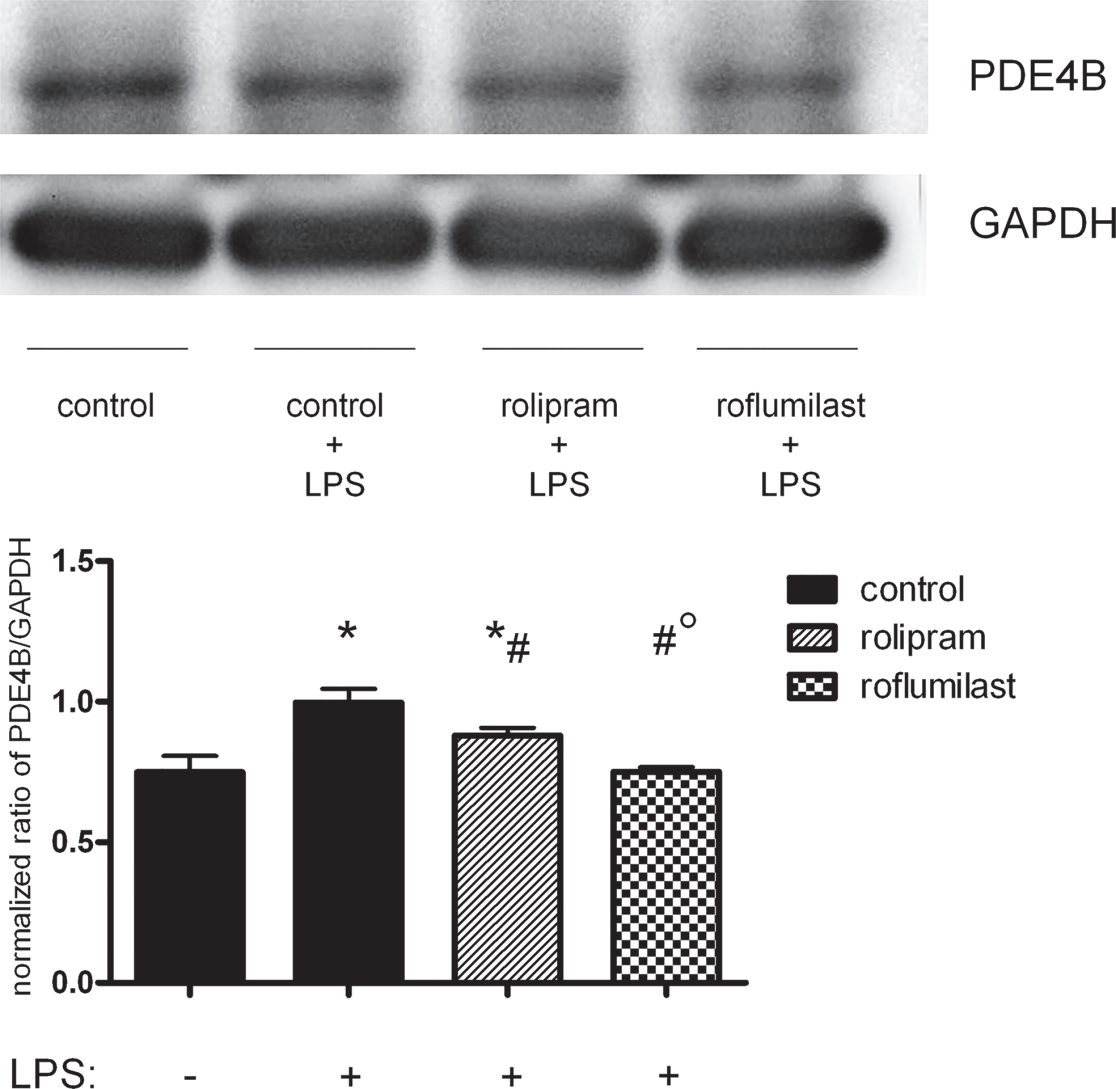
PDE4B protein expression in epithelial cells after LPS and rolipram/roflumilast treatment. To further confirm results from microscopy, we additionally performed western blots of the PDE4B of H441 cells. Images are representatives of four experiments with similar results (original magnification, x63). Data are presented as mean ± SD; n = 3; *P < 0.05 vs. control group without LPS inhalation, ^#^P < 0.05 vs. control with LPS; °P < 0.05 vs. the rolipram group.

**Fig 12:**
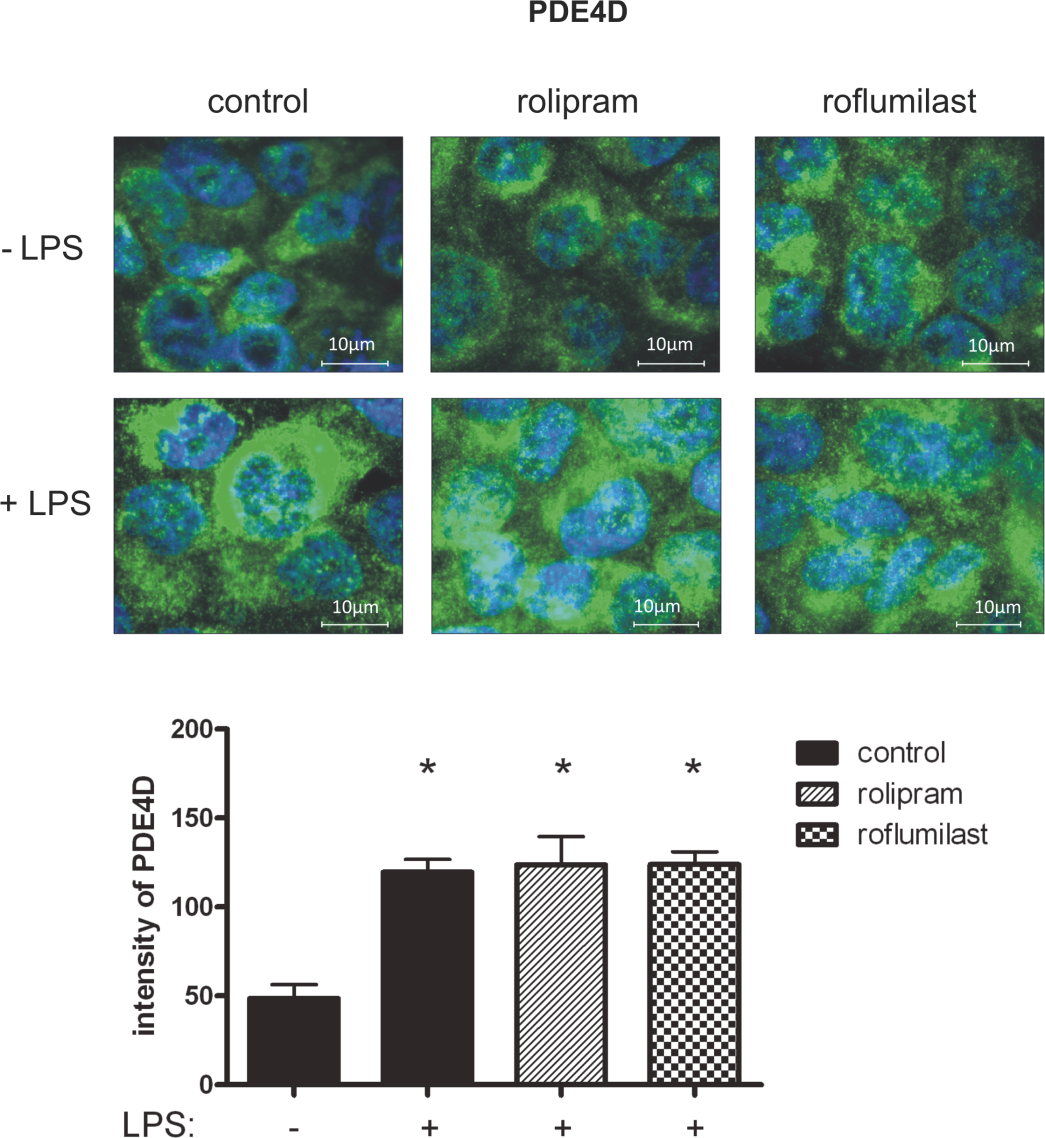
The effect of LPS and rolipram/roflumilast on PDE4D protein expression in epithelial cells. Human pulmonary epithelial cells were pretreated with rolipram/roflumilast and the influence of LPS on the expression of PDE4D (green) (**B.1**) evaluated. Images are representatives of four experiments with similar results (original magnification, x63). Data are presented as mean ± SD; n = 3; *P < 0.05 vs. control group without LPS inhalation, ^#^P < 0.05 vs. control with LPS.

**Fig 13:**
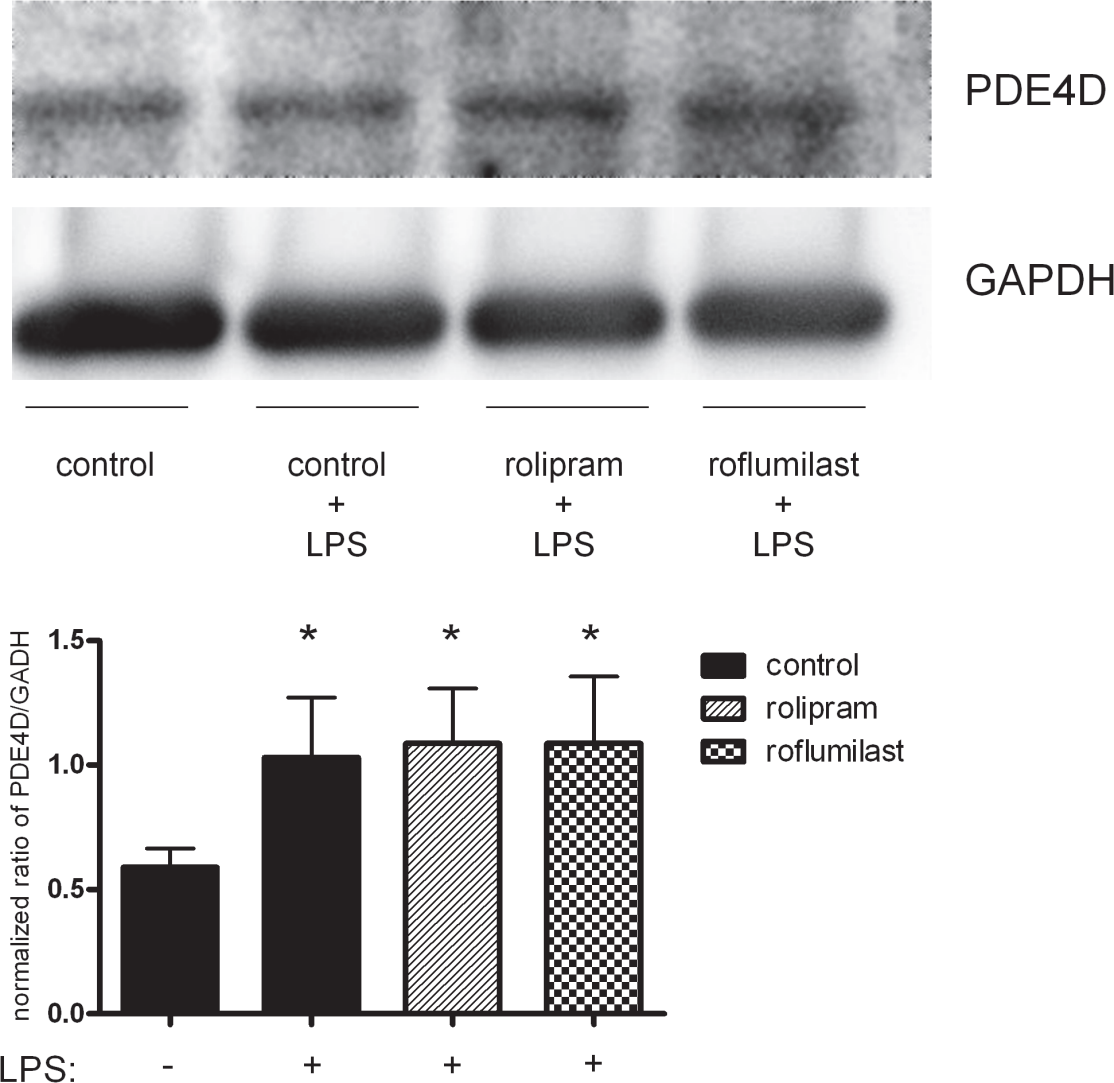
PDE4D protein expression in epithelial cells after LPS and rolipram/roflumilast treatment. To further confirm results from microscopy, we additionally performed western blots of the PDE4B of H441 cells. Images are representatives of four experiments with similar results (original magnification, x63). Data are presented as mean ± SD; n = 3; *P < 0.05 vs. control group without LPS inhalation, ^#^P < 0.05 vs. control with LPS.

**Fig 14:**
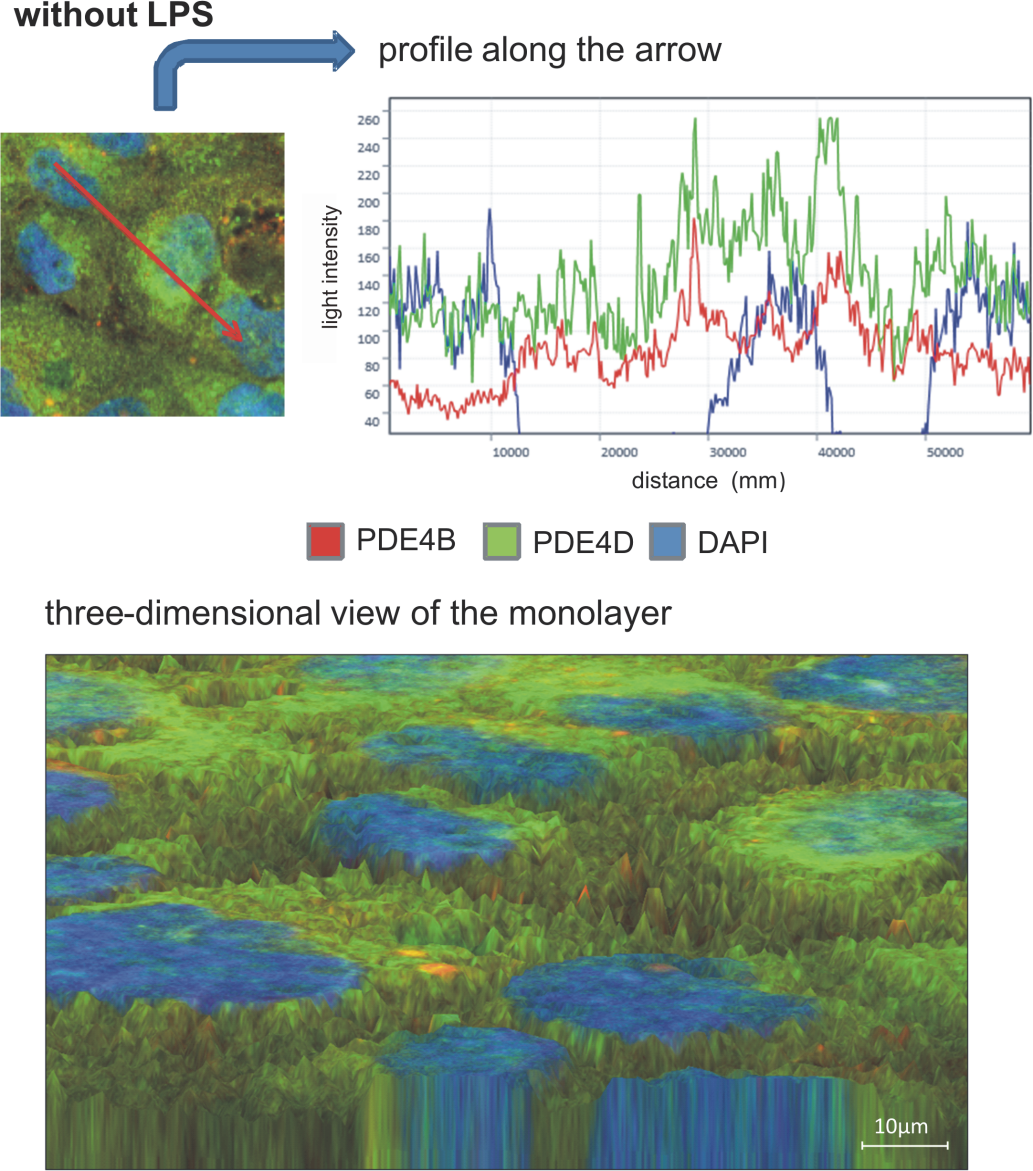
The distribution of PDE4B and PDE4D protein in epithelial cells. PDE4B and PDE4D were stained in one sample. Without LPS, PDE4D (green) is dominant. Both subtypes were found coexistent around the nucleus and also in the cytoplasm. Images are representatives of four experiments with similar results (original magnification, x63).

**Fig 15:**
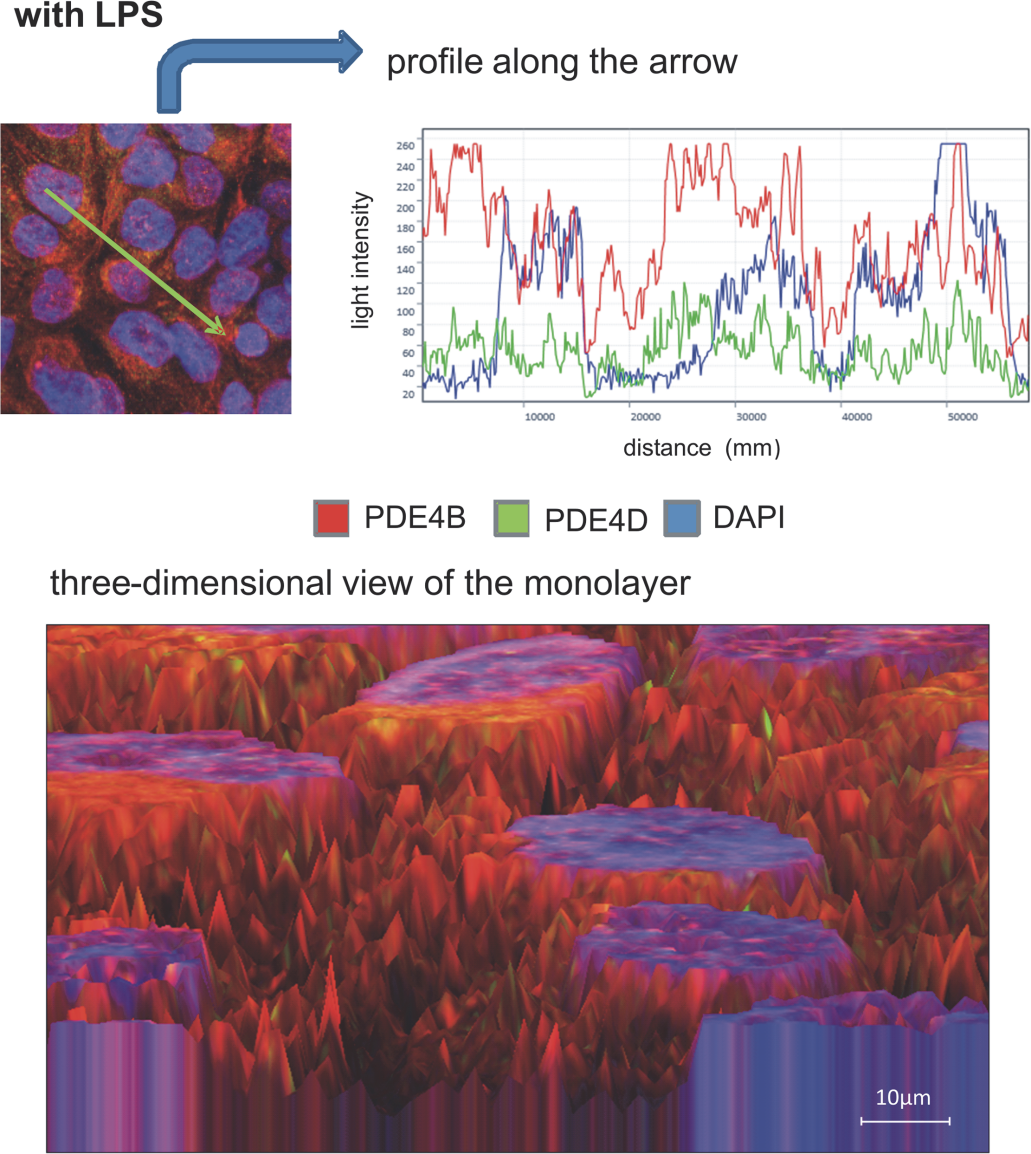
The influence of hyperinflammation on PDE4B and PDE4D protein expression in epithelial cells. PDE4B and PDE4D were stained in one sample to investigate the effect of LPS on the distribution of both enzymes. Even at first glance, it is striking that in the picture without LPS PDE4D (**Fig 14**) is dominant, whereas LPS increased predominantly PDE4B (red) (Fig 15), confirming our previous findings. Images are representatives of four experiments with similar results (original magnification, x63).

To further evaluate PDE4B and PDE4D on epithelial cells, we determined the distribution of the two enzymes in pulmonary epithelial cells with fluorescent staining. First, we investigated the impact of rolipram and roflumilast on the expression of the two subtypes of PDE4 individually and measured the light intensity of the enzymes. There were no differences in between the groups without an inflammation. LPS increased PDE4B protein in epithelial cells, whereas rolipram and to a higher extend roflumilast significantly reduced protein levels (roflumilast: 104±13; rolipram: 139±12; control: 171±12; all P < 0.05) (Fig 10), indicating the pivotal role of the PDE-inhibitors on epithelial cells. These findings were further confirmed by western blots (Fig 11) and further verify our data from gene and protein expression of PDE4B in the lungs of mice. LPS also enhanced PDE4D protein, but to a lesser degree as PDE4B (Fig 12). Rolipram and roflumilast did not alter PDE4D protein levels, which was also confirmed via western blots (Fig 13) (see S2 Fig for whole blots). In separate experiments, we stained PDE4B and PDE4D in one sample to investigate the effect of LPS on the distribution of both enzymes (Figs 14 and 15). Even at first glance, it is striking that in the picture without LPS PDE4D (green) is dominant, whereas LPS increased predominantly PDE4B (red), confirming our previous findings. Both subtypes were found coexistent around the nucleus and also in the cytoplasm.

## Discussion

We chose to investigate the impact of roflumilast compared to rolipram on the epithelium in LPS-induced pulmonary inflammation since roflumilast is already licensed for the use in humans and its precursor rolipram was used in most experimental studies so far. In the last years, the discussion in the literature pointed into investigating a selective PDE4B-inhibitor [14,28] to reduce the side effects associated with PDE4D-inhibition [10,29]. In 2013, Suzuki et al. brought new insight on this topic [30]. They compared a selective PDE4B compound with roflumilast in terms of the efficiency of reducing pulmonary neutrophilia, the reduction of TNFα, and gastric adverse effects. The impact of the selective PDE4B compound on pulmonary neutrophilia was weaker and finally, at higher concentrations of the compound and then comparable therapeutic effects, the specific PDE4B inhibitor was not superior related to gastric side effects compared to the unspecific inhibitor roflumilast. The authors still suggested that PDE4D is involved in gastric side effects of PDE4-inhibitors, since the inhibitory potency on gastric emptying of the specific compound was slighter.

The most feasible explanation for the weaker developed impact of the specific PDE4B compound on PMN migration is that PDE4D has also anti-inflammatory properties. This explanation is supported by the findings of Ariga et al., where PDE4D also interfered with PMN migration into the lung [9]. In their study, the administration of rolipram to PDE4B knockout mice still had an additional anti-inflammatory effect on PMN migration and, in addition, PDE4D knockout mice also showed reduced PMN counts after LPS-induced acute pulmonary inflammation. At first sight, this stays in contrast with studies that attributed only PDE4B an influence on LPS-induced TNFα production of monocytes and macrophages [7,8]. Suzuki et al. hypothesized that PDE4B predominantly inhibits TNFα production for chemotaxis, but for the inhibition of the actual PMN migration blocking both subtypes PDE4B and PDE4D is necessary [30]. In the presented study, we were able to ascribe the specific PDE4-inhibitors the influence on the reduction of gene and protein expression of PDE4B in the lungs of mice, while at the same time, not influencing the expression of PDE4D. We further determined the distribution of the PDE4B and PDE4D enzyme in pulmonary epithelial cells and were able to show the incremental role of PDE4B over PDE4D in LPS-stimulated epithelial cells. Rolipram, and even more intense roflumilast, reduced PDE4B expression, while not influencing PDE4D. The effect on protein levels can most probably be explained as temporary effect.

Further on, it can be hypothesized that roflumilast stabilizes pulmonal epithelial cells during inflammation better compared to rolipram due to decreased PDE4B. But in vivo and in vitro, LPS increased both subtypes. In contrast with our findings, nontypeable haemophilus influeznae (NTHi)-induced inflammation increased only PDE4B expression, but not PDE4D [31]. NTHi-induced inflammation acts via the TLR2 pathway, LPS via the TLR4 pathway. In the presented study, we detected an increase of PDE4D expression through LPS, which was also found by Gobjishvili et al. in primary hepatic stellate cells [32]. In our model, rolipram and roflumilast did not have any influence on PDE4D expression, suggesting that the side effects of the PDE4-inhibitors might depend on the signal cascade of the underlying disease causing the administration of the PDE4-inhibitors.

PDE4B is known to reduce TNFα levels [7,8,30], but most of the authors did not determine other chemokines than TNFα. In the presented study, we also detected the impact of roflumilast and rolipram on the potent chemoattractant TNFα and we further investigated and discovered their pivotal role in the reduction of the release of CXCL1, CXCL2/3, and IL-6. These chemokines are the most potent chemoattractants for neutrophils, and so far have not been investigated in terms of the use of PDE4-inhibitors. TNFα and IL6 induce neutrophil adhesion to the endothelium by increasing the adhesion molecules VCAM-1 (vascular cell adhesion molecule) and ICAM-1 (intercellular adhesion molecule) [33]. We did not detect any differences between the PDE4-inhibitors in terms of TNFα and IL6 reduction, which could explain why there were no differences concerning interstitial PMN counts between the two compounds. CXCL1 and CXCL2/3 are released in the BAL as the major chemoattractants for PMNs [34,35]. However, CXCL1 is not only expressed by neutrophils but also by the epithelium [35,36], highlighting the pivotal role of PDE-inhibitors on the epithelium. The lower CXCL1 levels in roflumilast treated animals explain and confirm our findings from the in vivo and in vitro transmigrationassay with a stronger effect of the compound in terms of reducing PMN migration transepithelial into the alveolar space.

Besides the reduction of CXCL1, an additional potential mechanism shown in the presented study might be the reduction of cytoskeletal remodeling by the PDE4-inhibitors, indicating their pivotal role in stabilization pulmonary barrier function. Underlying our findings of PDE-inhibitors influencing the epithelium, Moodley et al. determined the impact of PDE4-inhibitor in combination with inhaled corticosteroids and β_2_-adrenoreceptor agonists on pulmonary bronchial epithelium in terms of COPD in vitro [37]. PDE4-inhibitors further augmented the expression of glucocorticoid-induced genes in the epithelium.

In the presented study, roflumilast had a superior outcome on transepithelial PMN migration compared to rolipram. In relation to the second hallmark of acute pulmonary inflammation—microvascular permeability—we also detected a pivotal role of roflumilast on stabilization the capillary leakage. This last finding is in accordance with the findings of Sanz et al. [38], where roflumilast showed stronger effects in terms of E-selectin expression of stimulated endothelial cells and neutrophil CD11b expression, a cell surface marker required for transmigration.

The use of inhaled agents is well established in respiratory diseases to increase the local concentration of the compound associated with lower systemic concentrations and therefore weaker side effects. Recently, Jin et al. demanded the topical administration of PDE4-inhibitors as a potential to minimize side effects [5] and Zimmermann et al. detected that rolipram treatment significantly decreased epithelial microwound closure and defined rolipram as contraindicated for the use in chronic inflammatory bowel disorders [39]. To our knowledge, we are the first to demonstrate that nebulized rolipram and roflumilast have anti-inflammatory properties with the same effect on PMN migration. This finding highlights the clinical potential of roflumilast and has the potency to reduce gastrointestinal side effects. The other clinically important insight of the presented study is that roflumilast still showed anti-inflammatory effects even when given six hours after the inflammatory stimulus.

## Conclusions

In a LPS-induced model of pulmonary inflammation, PDE-inhibitors prevented transendothelial and transepithelial PMN migration into the different compartments of the lung. The predominant effect of roflumilast on stabilizing the alveolocapillary barrier was also pivotal on pulmonary epithelium. LPS inhalation induced the expression of PDE4B and PDE4D, but PDE-inhibitors reduced the inflammatory PDE4B and only slightly affected the mainly emetic-acting PDE4D. Furthermore, we highlighted the clinical impact of roflumilast since it still showed anti-inflammatory properties when administered after the inflammatory stimulus, and additionally topical administration as a possibility to reduce side effects has also been effective.

## Supporting Information

S1 Fig Whole western blots of cropped bands of PDE4B, PDE4D and GAPDH shown in Fig 6. (TIF)

S2 Fig Whole western blots of cropped bands from Fig 10A.2 and 10B.2 of PDE4B and PDE4D expression in pulmonary epithelial cells. 1 = control; 2 = control+LPS; 3 = rolipram+LPS; 4 = roflumilast+LPS.
(TIF)
