# Supplementary figures and images for: The Unrecognized Effects of Phosphodiesterase 4 on Epithelial Cells in Pulmonary Inflammation
Source: PLoS One. 2015 Apr 24;10(4):e0121725. doi: 10.1371/journal.pone.0121725 (PMC4409344; doi:10.1371/journal.pone.0121725)

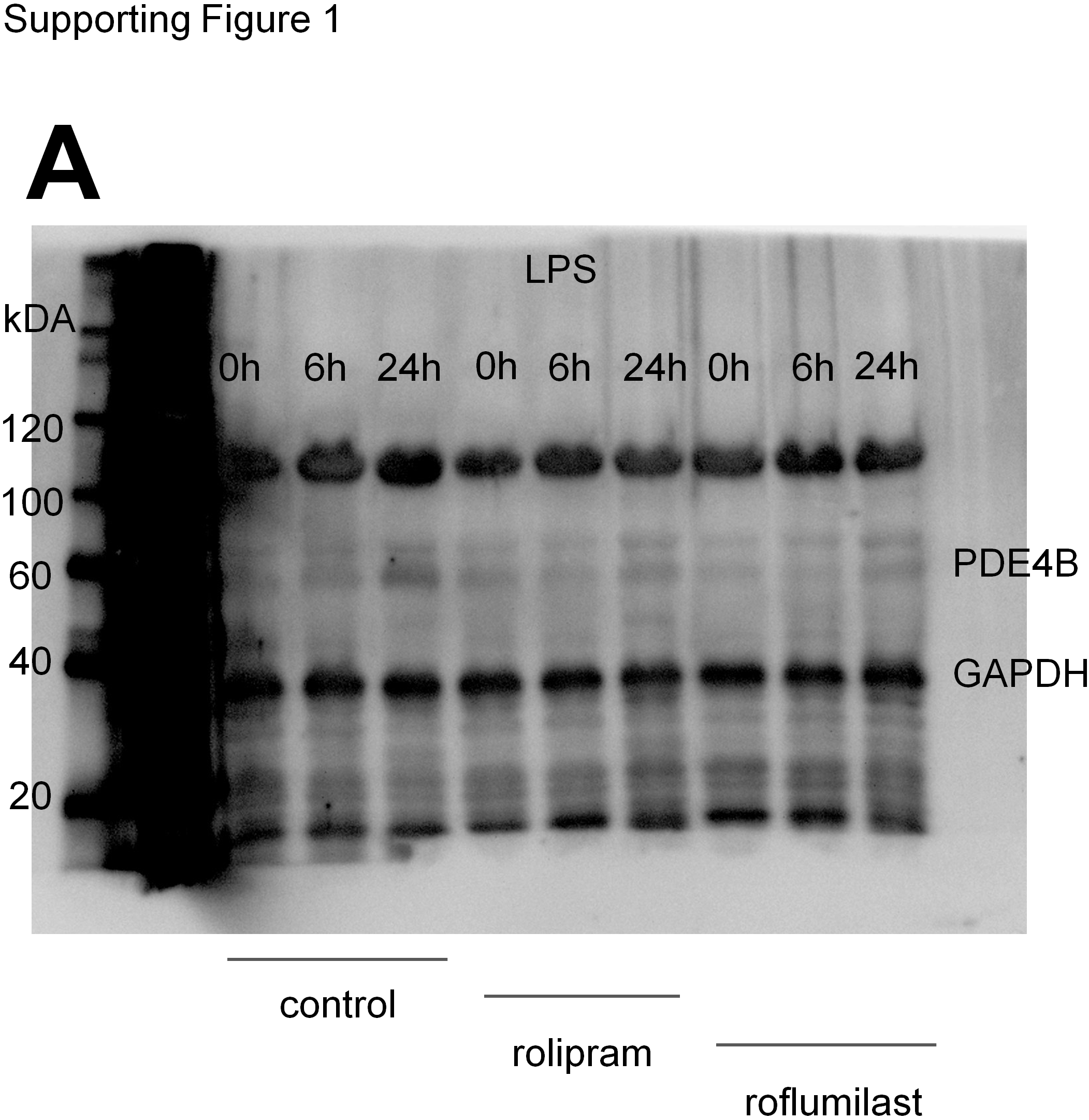

Supplement: S1 Fig — (TIF) [file pone.0121725.s001.tif]

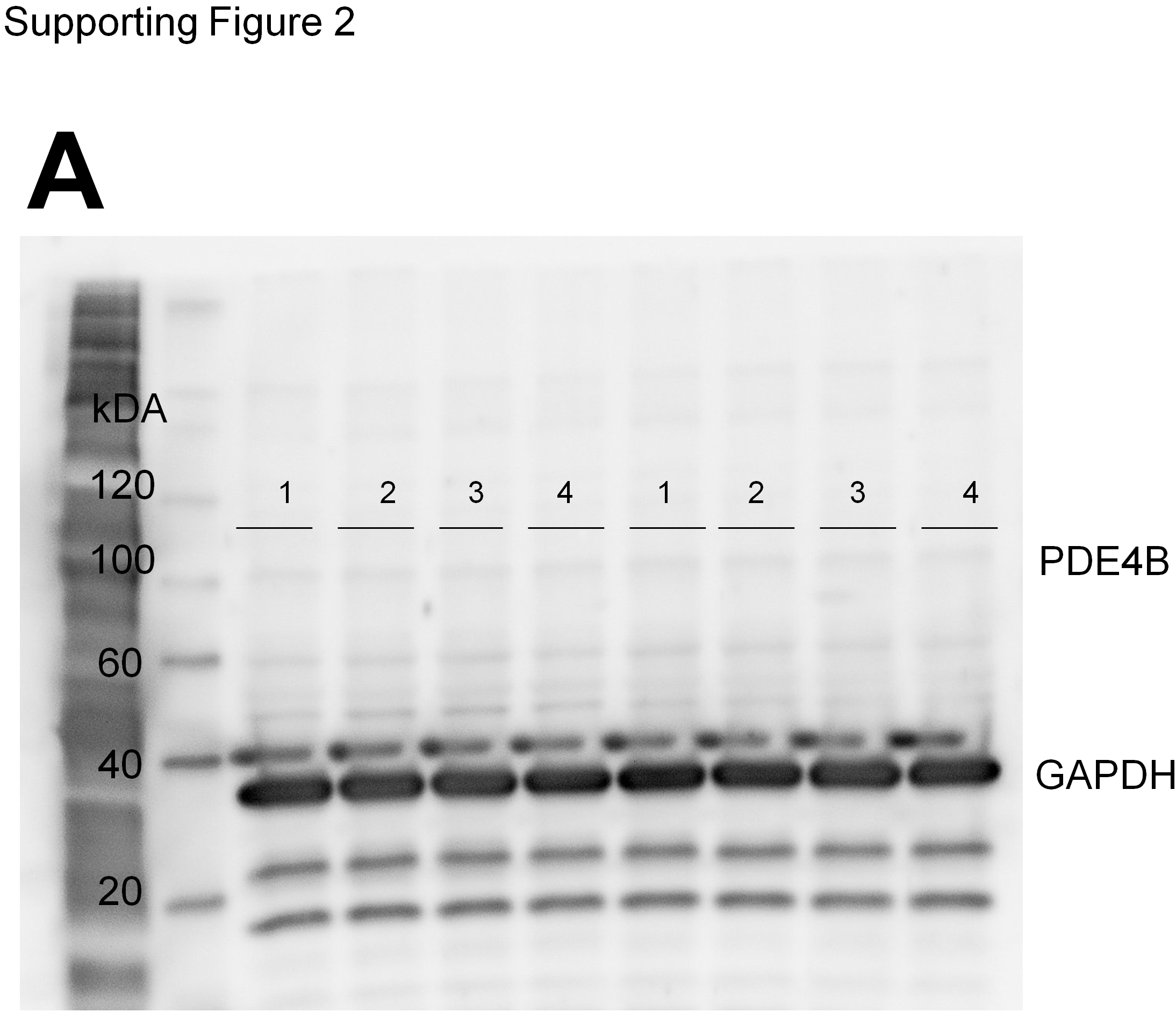

Supplement: S2 Fig — 1 = control; 2 = control+LPS; 3 = rolipram+LPS; 4 = roflumilast+LPS. (TIF) [file pone.0121725.s002.tif]
